# Delineation of Molecular Lesions in Acute Myeloid Leukemia Patients at Diagnosis: Integrated Next Generation Sequencing and Cytogenomic Studies

**DOI:** 10.3390/genes12060846

**Published:** 2021-05-30

**Authors:** Sorina Mihaela Papuc, Alina Erbescu, Diana Cisleanu, Diana Ozunu, Cristina Enache, Ion Dumitru, Elena Lupoaia Andrus, Mihaela Gaman, Viola Maria Popov, Maria Dobre, Oana Stanca, Silvana Angelescu, Nicoleta Berbec, Andrei Colita, Ana-Maria Vladareanu, Horia Bumbea, Aurora Arghir

**Affiliations:** 1 Victor Babes National Institute of Pathology, 050096 Bucharest, Romania; ela.papuc@ivb.ro (S.M.P.); erbescua@gmail.com (A.E.); diana.ochiana@gmail.com (D.O.); maria_dobre70@yahoo.com (M.D.); 2 Carol Davila University of Medicine and Pharmacy, 050474 Bucharest, Romania; dcisleanu@yahoo.com (D.C.); c.t.enache@gmail.com (C.E.); elenaandrus@yahoo.com (E.L.A.); mihaela_dervesteanu@yahoo.com (M.G.); oacioc@yahoo.com (O.S.); sangelescu21@gmail.com (S.A.); nicole_olt@yahoo.com (N.B.); andreicolita@yahoo.com (A.C.); anamariavladareanu@yahoo.com (A.-M.V.); horiabum@gmail.com (H.B.); 3 Emergency Universitary Clinical Hospital, 050098 Bucharest, Romania; punctdoc@yahoo.com; 4 Colentina Clinical Hospital, 020125 Bucharest, Romania; violamariap@gmail.com; 5 Coltea Clinical Hospital, 030167 Bucharest, Romania

**Keywords:** somatic mutations, copy number variants, chromosomal abnormalities, mutational screening, detection yield

## Abstract

Acute myeloid leukemia (AML) is a heterogeneous disorder characterized by a wide range of genetic defects. Cytogenetics, molecular and genomic technologies have proved to be helpful for deciphering the mutational landscape of AML and impacted clinical practice. Forty-eight new AML patients were investigated with an integrated approach, including classical and molecular cytogenetics, array-based comparative genomic hybridization and targeted next generation sequencing (NGS). Various genetic defects were identified in all the patients using our strategy. Targeted NGS revealed known pathogenic mutations as well as rare or unreported variants with deleterious predictions. The mutational screening of the normal karyotype (NK) group identified clinically relevant variants in 86.2% of the patients; in the abnormal cytogenetics group, the mutation detection rate was 87.5%. Overall, the highest mutation prevalence was observed for the *NPM1* gene, followed by *DNMT3A, FLT3* and *NRAS*. An unexpected co-occurrence of *KMT2A* translocation and *DNMT3A*-R882 was identified; alterations of these genes, which are involved in epigenetic regulation, are considered to be mutually exclusive. A microarray analysis detected CNVs in 25% of the NK AML patients. In patients with complex karyotypes, the microarray analysis made a significant contribution toward the accurate characterization of chromosomal defects. In summary, our results show that the integration of multiple investigative strategies increases the detection yield of genetic defects with potential clinical relevance.

## 1. Introduction

Acute myeloid leukemia (AML) is a clonal hematopoietic neoplasm characterized by the uncontrolled proliferation and accumulation of leukemic blasts. AML is not a single clinical entity, but rather a heterogeneous group of disorders with complex biology, molecular heterogeneity and variable treatment outcomes [1,2,3,4]. 

AML architecture is, as in many other cancers, the result of a multi-step process with the successive accumulation of molecular lesions; a complex interplay of different types of driver events, from gross chromosomal anomalies to single nucleotide variants, has been described in AML. The molecular heterogeneity of AML has long been recognized since the early observations of different cytogenetic changes, such as t(8;21)(q22;q22) and t(15;17)(q24;q21) [5,6]. The identification of various recurrent chromosomal rearrangements in AML paved the way for AML gene discovery, toward a better understanding of AML molecular pathogenesis. Recent technological advances, such as chromosomal microarrays (CMAs) and the next generation sequencing (NGS) of gene panels, whole exome or whole genome, have allowed progressive unravelling of the molecular landscape of AML with important consequences for clinical practice. 

As none of the existing genetic technologies enable the straightforward detection of all the genetic defects in a clinical AML sample, combinations of different complementary assays are currently used. Classical karyotyping interrogates the entire genome at the chromosomal level, enabling the detection of balanced and unbalanced structural rearrangements as well as numerical aberrations. Moreover, by describing the chromosomal constitution of each cell, cytogenetics is an excellent tool for deciphering the clonal architecture and clonal evolution of AML [7,8,9]. FISH tests used in combination with karyotype analysis brings the benefit of increased resolution and specificity. In addition, interphase FISH is a fast assay for the detection of recurrent chromosomal anomalies, which is especially useful for samples with no dividing cells [10]. The current recommendations for diagnostic workup include routine FISH screening for chromosomal anomalies with a high impact on clinical management known to be frequently missed by routine classical karyotyping (e.g., *KMT2A* and *MECOM* gene rearrangements) [11,12]. Classical and molecular cytogenetic analysis of leukemic cells allows for accurate diagnostic classification and provides important, independent prognostic information [7,8,9,13]. However, as ~40–50% of AML patients have normal karyotypes [10,14], molecular genetic investigations are mandatory [12].

High-resolution, genome-wide technologies, such as array-based comparative genomic hybridization (Array-CGH), SNP microarrays and hybrid microarrays (Array-CGH+SNP), generically named CMA, offer substantial advantages over classical karyotyping and FISH tests. These technologies allow for the molecular characterization of genomic imbalances, providing valuable information, such as accurate size, gene content, and breakpoints, for both cytogenetically visible and cryptic aberrations, otherwise undetectable [15,16,17,18]. SNP-based microarray platforms are gold-standard technologies that enable the genome-wide detection of copy neutral loss of heterozygosity (CN-LOHs), considered relevant for cancer pathogenesis [19]. The wide use of CMA for the assessment of genomic imbalances and CN-LOH revealed their clinical utility for prognostic stratification [18,20]. This is particularly true for AML patients with normal cytogenetics and cases with failed cytogenetic results (~10% of all cases) for whom CMA can provide valuable information for risk assessment. [10,20,21,22,23]. In addition, in relapsed AML, CMA proved useful for detecting CNVs that contribute to resistance to chemotherapy, such as the deletion of *TP53* gene and the amplification of *ERG* locus [20]. 

NGS is a powerful molecular tool, allowing the parallel sequencing of multiple targets and the detection of clinically relevant variants. Recently, large sequencing studies have dissected the AML genome and established comprehensive catalogues of AML genes and their respective mutational frequencies [3,4]. NGS became a driving force in the field of gene discovery and characterization of clonal architecture. Targeted NGS is increasingly incorporated into the clinical routine, due to its versatility, ability to screen a high number of genes and capacity to detect different types of molecular defects [9,13,24,25,26,27]. Mutational screening by targeted NGS with comprehensive gene panels detected at least one driver mutation in > 90% of patients [3,4]. NGS makes an excellent contribution to the diagnostic classification and prognostic stratification of AML with normal karyotype, but also for AML with recurrent genetic changes. Last but not least, NGS has the potential to rapidly screen for and unravel molecular lesions that are now targetable with specific therapeutic strategies, thus is an important tool for personalized approaches [9,13,24].

With the combined use of classical genetic techniques and increasingly powerful genomic technologies, the characterization of AML patients’ genomes is incorporated into the routine workup, leading to a better diagnosis, patient stratification and therapeutic guidance [9,11,12,24]. 

Comprehensive AML diagnosis has a major impact on clinical care. The diagnostic classification systems in AML evolved progressively from morphological description- based to genetically informed systems [1,4,9,28]. In 2001, the WHO classification included four cytogenetically defined diagnostic entities [29]. The 2008 revision extended to seven the number of AML cytogenetic subtypes and recognized, for the first time, gene mutations as entity-defining lesions (provisional AML with mutated *NPM1* and AML with mutated *CEBPA)* [30]. In the most recent revision of the WHO classification, one provisional and two definitive entities are defined by specific sequence variants (*RUNX1, NPM1* and bi-allelic *CEBPA*, respectively) based on the accumulating evidence that supports the role of gene mutations in AML biology [11]. 

Prognostic evaluation of AML at diagnosis is another critical step in clinical management. Genetic risk stratification, according to the European Leukemia Net (ELN) 2017 recommendations, is based on cytogenetic data and the mutational screening of *NPM1, FLT3, RUNX1, ASXL1* and *TP53.* There are three risk categories: favorable, intermediate and adverse. t(8;21), inv(16), and *NPM1* gene mutations (with low *FLT3*-ITD mutant/wild-type allelic ratio or without *FLT3*-ITD) and bi-allelic *CEBPA* mutations fall within the favorable risk category. Six cytogenetic scenarios and four gene mutation events *(ASXL1*, *RUNX1, TP53* and high *FLT3*-ITD with wild type *NPM1*) are associated with adverse risk. The intermediate risk category is highly heterogeneous and includes several cytogenetic and molecular defects not included in the previous two risk categories [11]. 

Accurate WHO classification and prognostic evaluation according to the current ELN recommendations require integrated strategies of hematological, cytogenetic and molecular studies [9,11,12,24,31]. The recommendations for genetic testing are constantly updated in order to incorporate new, optimized techniques and provide a robust and efficient clinical laboratory routine [11,12,24]. The AML multidisciplinary diagnostic approach is also expected to bring an increase in knowledge that will better inform prognostic evaluation and therapeutic guidance. In the context of recent advances in AML treatment and the approval of new drugs, individualized treatment will be increasingly assisted by comprehensive molecular diagnostic [2,9,24,32]. 

The aim of our study was to better characterize a group of 30 normal cytogenetics AML patients, using NGS and Array-CGH+SNP, with the purpose of describing the spectrum of recurrent mutations in AML genes and discovering new variants of potential interest for the disease. In addition, for a set of 16 AML patients with abnormal cytogenetics, the abovementioned genomic technologies were used for molecular definition of chromosomal anomalies. By comparing the detection yield and contribution of each genetic technique to the description of the genomic landscape within our patient group, we tested the hypothesis that the integrated use of NGS and Array-CGH+SNP, in addition to classical cytogenetic and genetic testing increases the detection rate of clinically relevant molecular lesions.

## 2. Materials and Methods

### 2.1. Patients

Forty-eight patients were enrolled with a diagnosis of AML. At presentation, the patients were classified as having de novo AML (84%; 40/48), AML with myelodysplasia-related changes (10%; 5/48) or therapy-related AML (6%; 3/48).

The routine diagnostic workup consisted of bone marrow and/or peripheral blood morphological evaluation, flow-cytometry, classical cytogenetics and fluorescent in situ hybridization (FISH) tests, using standard protocols. Karyotype analysis was performed on 20 metaphases, when available; ISCN 2016 recommendations were followed for karyotype description. 

Patients were enrolled for further molecular characterization if classified in the normal karyotype (NK) group or if they had intermediate and adverse cytogenetics according to ELN recommendations [9]. 

The research workup included FISH tests, array-CGH+SNP, PCR-based assays and capillary electrophoresis for FLT3-ITD testing, and targeted NGS. 

FISH tests were performed on metaphase chromosomes, using whole chromosome painting (WCP), centromeric (CEP), locus specific and multiprobe FISH probes according to the manufacturer’s recommendations. FISH was applied for molecular characterization of chromosomal anomalies, mostly in cases with complex karyotype or low banding resolution, and for CNVs confirmation or further analysis of genomic architecture in 29 patients. The selected locus-specific FISH probes were: *MLL (KMT2A*, 11q23) (Cytocell, OGT Cambridge, United Kingdom; Vysis, Abbott Laboratories, Abbott Park, IL, USA; Kreatech, Leica, Amsterdam, The Netherlands), *EVI1* (*MECOM*, 3q26) (Cytocell), *NUP98* (11p15) (Kreatech), and *CUX1/EZH2*/CEN 7 (7q22, 7q36, CEP7) (ZytoVysion, Bremerhaven, Germany). Dual Fusion, Dual Color Translocation probes for *CBFB/MYH11* [inv(16)(p13.1q22)/t(16;16)(p13.1;q22)] (Cytocell), *PML/RARA* [t(15;17)(q24;q21.1)] (Cytocell), *BCR/*ABL1 [t(9;22)(q34;q11.2)] (Cytocell), and *RPN1/MECOM* [t(3;3)(q21.3;q26.2)/inv (3)(q21.3q26.2)] (Kreatech-Leica), the centromeric probes CEPX, CEP1, CEP6, CEP8, CEP9, and CEP11 (Cytocell), the whole chromosome painting WCP3, WCP7, WCP8, WCP9, WCP11, WCP12, WCP21 (Cytocell), WCP8 (Kreatech), and Multiprobe Octochrome (Cytocell) were used. 

### 2.2. Genomic DNA (gDNA) Extraction

gDNA extraction from diagnostic whole bone marrow (46 patients) and peripheral blood (2 patients) samples was performed with a PureLink Genomic DNA Mini Kit (Thermo Fisher Scientific, Waltham, MA, USA) according to the manufacturer’s recommendations with minor changes. The quality and quantity of the qDNA were assessed using Nanodrop 2000 spectrophotometer and Qubit 2.0 Fluorometer with the DNA Broad Range assay Kit (Thermo Fisher Scientific). High purity gDNA samples, with A260/280 = 1.8–2 and A260/230 > 1, were used for the subsequent molecular investigations, when available. 

### 2.3. Genomic Profiling

The array-CGH+SNP was performed using the SurePrint G3 Cancer CGH+SNP Microarray Kit 4x180K (Agilent Technologies, Santa Clara, CA, USA), containing 110,712 CGH and 59,647 SNP probes, with an overall median spacing of 25 kb, annotated on NCBI Build 37. The recommendations of the Agilent Enzymatic labeling protocol for blood, cells and tissue protocol (version 7.5) were followed. In brief, 500–1000 ng of gDNA was digested with *Alu*I and *Rsa*I, and subsequently labeled with cyanine 5 deoxyuridine triphosphate (Cy5-dUTP) (reference DNA) and Cy3-dUTP (patient DNA), using the SureTag DNA Labeling Kit (Agilent Technologies). Commercial human gDNA was used as a reference (Agilent Human Reference DNA male or female, according to the sex of the patient). The hybridization took place at 67 °C for 24 h with constant rotation (20 rpm). Immediately after the post-hybridization washes, the oligonucleotide slides were scanned with the Agilent SureScan Microarray Scanner System; Agilent Cytogenomic Software v5.1.2.1 was used for raw data extraction and data analysis (Agilent Technologies). Diploid peak centralization and GC correction were used for normalization. The aberration detection method-2 (ADM2) algorithm (Agilent Technologies) was used for CNV detection. In order to be called, the CNVs had to cumulatively fulfil the following criteria: size over 100 kb, a uniform probe profile on visual inspection and a log2 ratio value over 0.3 or below −0.3. CN-LOH regions larger than 10 Mb were considered for further analysis. For the clinical interpretation of genomic variants, our data were compared with those from the literature and public databases: UCSC (http://genome.ucsc.edu, last accessed on 27 April 2021); DGV (http://dgv.tcag.ca/dgv/app/, last accessed on 28 April 2021), OMIM (http://www.omim.org/, last accessed on 29 April 2021), and COSMIC (https://cancer.sanger.ac.uk/cosmic, last accessed on 29 April 2021). Polymorphic regions with copy number aberrations reported in the general population (DGV) were excluded from further analysis. 

### 2.4. FLT3-ITD Screening

PCR followed by fragment analysis by capillary electrophoresis (ABI 3500 Genetic Analyzer) was performed for *FLT3-ITD* testing. The PCR was performed starting from 50 ng of gDNA in a total reaction volume of 25 μL, containing the following: 1U of Taq DNA Polymerase, 0.5 μL dNTP mix (10 mM), 1× PCR Buffer, 2 mM MgCl_2_, and 0.5 μM of each primer. The primers for ITD detection were fluorescently labeled: 5′-HEX-GCAATTTADGTATGAAAGCCAGC-3′ (forward), and 5′-FAM-CTTTCAGCATTTTGACGGCAACC-3′ (reverse) [33]. The cycling conditions were as follows: denaturation for 3 min at 94 °C; 40 cycles of 45 s at 94 °C, 60 s at 61°C, 90 s at 72 °C; and final extension for 15 min at 72 °C. The amplification products were denatured with Hi-Di Formamide and migrated on ABI 3500 Genetic Analyzer (Thermo Fisher Scientific), using the GeneScan 600 LIZ Standard (Thermo Fisher Scientific) for size evaluation. The data were analyzed with the GeneMapper Software v5 (Thermo Fisher Scientific); the amplicons larger than the expected wild type (330 bp) were interpreted as positive for the ITD variant.

### 2.5. Ion Torrent Targeted NGS 

Targeted NGS testing was performed for 45 patients, using the Ion AmpliSeq™ AML Research Panel, which covers 19 genes, on the Ion PGM System (Thermo Fisher Scientific). The panel generated 237 amplicon sets covering the entire coding sequences of 5 genes (*CEBPA, DNMT3A, GATA2, TET2,* and *TP53*) and hotspot regions of 14 genes (*ASXL1, BRAF, CBL, FLT3, IDH1, IDH2, JAK2, KIT, KRAS, NPM1, NRAS, PTPN11, RUNX,* and *WT1*). The libraries were prepared using the Ion AmpliSeq Library Kit 2.0 starting from 10 ng of gDNA /pool (4 pools/sample) under conditions specified by the manufacturer. After the barcode-adapters attachment for each sample (Ion Xpress Barcode Adapters 1-16 kit, Thermo Fisher Scientific) and AMPure XP purification (Beckman Coulter, Brea, CA, USA), the libraries were further amplified, purified and quantified (Qubit dsDNA HS Assay Kit, Thermo Fisher Scientific) according to the manufacturer’s protocol.

The Ion One Touch 2 Instrument was used for emulsion PCR with Ion PGM Hi-Q OT2 and Ion PGM Hi-Q View OT2 Kits, followed by target enrichment of Ion Sphere Particles (ISPs) on the Ion One Touch ES Instrument (Thermo Fisher Scientific). The Ion Sphere Quality Control Kit (Thermo Fisher Scientific) was used for the quality assessment of the enrichment, following manufacturer’s instructions. The positive IPSs were loaded on Ion 318 v2 chips using 500-flow runs and sequenced on Ion Torrent PGM instrument (Thermo Fisher Scientific) as recommended. 

Sequence alignment and analysis were performed using the Ion Reporter software v.5.10.1. (Thermo Fisher Scientific), Variant Caller plugin v.5.0.4.0 (Thermo Fisher Scientific), and NextGene software v.2.4.2 (SoftGenetics, State College, PA, USA). Raw data were aligned to the human genome build 19 (hg19, GRCh37) and annotated using dbNSFP v.2.9., dbSNP and the COSMIC database (https://cancer.sanger.ac.uk/cosmic/gene/, accessed on 12 April 2021). The average sequencing coverage was 1220 with >91% of the region-of-interest covered at least 100-fold. Non-synonymous and splice-site variants (12 intronic and 3 coding base-pairs) with a variant allele frequency (VAF) ≥ 2 % were called; the silent, 5′ and 3′ untranslated regions (UTRs), far intronic variants, and polymorphisms with a minor allele frequency (MAF) in the general population of more than 1% were excluded from analysis. We classified as pathologic or likely pathologic, recurrent or rare variants reported in the COSMIC and ClinVar databases or unreported variants without a MAF score and with deleterious predictions from standard in silico prediction tools, such as SIFT (http://sift.jcvi.org/, accessed on 16 April 2021), PolyPhen2 (http://genetics.bwh.harvard.edu/pph2/, accessed on 16 April 2021), LRT, MutationTaster (http://www.mutationtaster.org, accessed on 16 April 2021), MutationAssessor, FATHMM, GERP and CADD (http://cadd.gs.washington.edu/, accessed on 16 April 2021).

## 3. Results and Discussion

We used an integrated genetic profiling approach that included classical cytogenetics, FISH, array-CGH+SNP, and targeted NGS for the molecular characterization of a selected group of 48 patients. Classical karyotyping was performed for all, except two cases with no available metaphases. These two patients were, however, characterized by array-CGH+SNP and NGS. The patients with available cytogenetics (46) were further analyzed by NGS (43) and array-CGH+SNP (36). Thus, in our study group, each patient was analyzed by a combination of at least two methods, and 34 patients were tested by cytogenetics, array-CGH+SNP and targeted NGS.

### 3.1. Cytogenetic Testing

Classical cytogenetic analysis revealed chromosomal anomalies in 35% (16/46) and no visible changes in 65% (30/46) of our patients. Previous studies showed that approximately half of AML cases have no visible changes on karyotype examination [13,34]. The distribution of our patients according to cytogenetic findings is different from that reported in the literature due to the patients’ selection methods used for this study. 

Sixteen patients had abnormal cytogenetic findings classified in the genetic adverse risk category (10 patients) and intermediate risk category (six patients). Adverse risk cytogenetics comprised complex karyotype changes (CK) (nine patients) and t(11;11)(p15;q23) with *NUP98-KMT2A* gene fusion (one patient). CK was defined as the presence of ≥three unrelated chromosomal abnormalities in the absence of a WHO recurrent translocation or inversion [35]. The degrees of complexity varied in our CK patients, from three to more than 10 independent anomalies (Supplementary Materials Table S1). The classification into the CK category was based on classical cytogenetics findings only for all the patients. FISH testing played an essential role in deciphering the chromosomal origin of the fragments involved in structural anomalies, especially in unbalanced translocations. Classical karyotyping and standard FISH were not sufficient for understanding very complex rearrangements (patient 47, Supplementary Materials Table S1). As reported in other cytogenetic studies of AML cohorts, classical karyotyping alone can only partially decipher CK. In most cases, additional molecular testing is required, especially for the characterization of derivative chromosomes generated through complex unbalanced translocations or other structural rearrangements, and marker and ring chromosomes description [7,35,36].

In our group, chromosome 5 was the most frequently altered chromosome in CK cases (7/9), followed by chromosome 8 (6/9) and chromosomes 7, 12 and 16 (4/9 each), similar to previous reports [7,35]. All of our CK patients had coexisting numerical and structural abnormalities; for the latter, the vast majority were unbalanced rearrangements.

Intermediate risk cytogenetic changes consisted of recurrent anomalies, such as t(9;11)(p21.3;p23.3)(*MLLT3*-*KMT2A*), t(1;19)(p13;p13.1) with an extra derivative chromosome 1, der(1)t(1;19), trisomy 4, trisomy 13, and trisomy 21, and one non-recurrent, complex translocation involving chromosomes 9 and 11 (patient 29, Supplementary Material Table S1). 

No visible chromosomal abnormalities were found in 30 patients (normal karyotype—NK). 

### 3.2. Genomic Profiling

The array-CGH+SNP results were available for 38 AML patients; 36 of them also had cytogenetic data. Genomic profiling was performed for 24 NK patients (24/30). In the adverse and intermediate genetic risk categories, eight (8/10) and four (4/6) patients, respectively, were investigated by microarray. 

#### 3.2.1. Array-CGH+SNP in NK Group 

The assessment of array-CGH+SNP benefits in AML investigation started with data analysis for the NK patient group. CNVs involving known AML genes/regions and putative CNVs were considered for further analysis. Large CN-LOHs previously reported in AML or encompassing cancer genes were also analyzed. Overall, nine CNVs and nine CN-LOH regions were detected. The detection yield of microarrays in NK AML group was 25% (6/24) for CNVs and 29% (7/24) for CN-LOH. This is in line with previous studies which reported detection rates ranging from 24% [3,17] to 58% [22] for CNVs and from 19% [37] to 32% [38] for CN-LOH. The CNVs involved nine different genomic regions distributed on chromosomes 5q, 7q, 11p, 11q, 12p, 15q, and 19p. The size of the genomic imbalances varied from 21.6 kb to 13 Mb, with a median of 503 kb. With the exception of one CNV, an intragenic duplication of *KMT2A* with a size of ~21 kb spanning exons 2 to 10 interpreted as a partial tandem duplication, all the CNVs were > 200 kb. CN-LOH regions had a median size of 47.3 Mb (13.3–120 Mb) and were distributed across chromosomes 1p, 2q, 3q, 6p, 7q, 11q, and 13q. CN-LOHs in these regions were previously reported in NK AML [22,38,39]. In our NK patients, CN-LOH led to a loss of heterozygosity for the *FLT3* mutation and *KMT2A*-PTD, each in one patient. 

**Figure 1:**
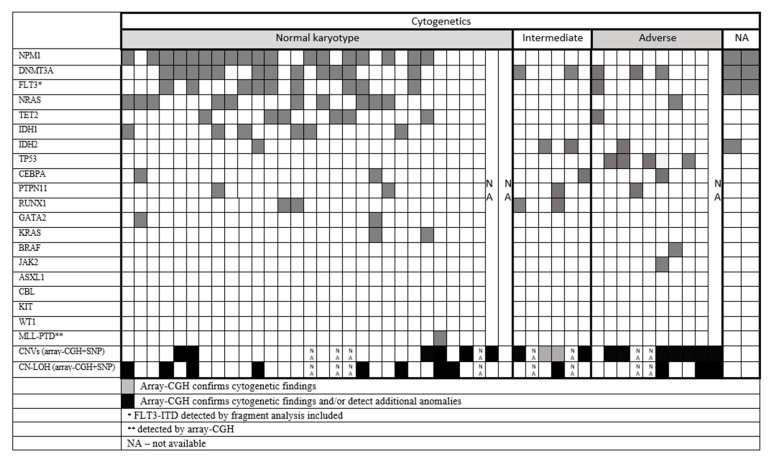
Summary of the genetic results illustrating the cooccurrences of different types of molecular defects.

Array-CGH+SNP testing was highly informative for two patients with no chromosomal changes and no mutations found on sequencing screening (Figure 1). One had a large region of CN-LOH spanning 54.7 Mb on chromosome 3q24q29 (genomic coordinates on hg19, chr3:142924211_197709783). This region includes 10 oncogenes, one of them being *MECOM*, and 12 tumor suppressor genes. The other had a 21 kb intragenic focal duplication spanning exons 2–10 of the *KMT2A* gene and a large CN-LOH (46.5 Mb) that included *KMT2A* intragenic duplication. In addition, two terminal CNVs were detected, suggestive of an unbalanced translocation: one duplication 11q24.1q25 and one deletion 12p13.33p13.2. With the use of WCP probes for chromosomes 11 and 12, an unbalanced cryptic translocation was unveiled. A breakapart *KMT2A* probe had a normal fluorescent pattern, thus making array-CGH+SNP the only genetic technique capable of unravelling the genomic defects of this patient. For two other patients from the NK group, microarray investigation identified CNVs involving AML critical regions (deletion 5q32, patient 13, Supplementary Materials Table S1) or genes (*CUX1*, patient 16, Supplementary Materials Table S1) [40]. 

#### 3.2.2. Array-CGH+SNP in the Intermediate Cytogenetics Group 

In the intermediate cytogenetics group, nine CNVs and two CN-LOH regions were detected. Array-CGH+SNP confirmed the cytogenetic findings in all tested patients (4) and added new findings: CNVs undetectable by classical karyotyping (two patients) and two CN-LOH regions (one patient). 

#### 3.2.3. Array-CGH+SNP in the Adverse Risk Cytogenetics Group 

In the CK AML group, the genomic profiling of seven out of nine patients was also informative, revealing a high burden of CNVs. The median number of CNVs per patient was 10, with the higher limit of the interval being 19. Virtually all the chromosomes were described at least once in the CK abnormalities, by classical cytogenetics and/or array-CGH+SNP, except for chromosomes 2, 14, 20 and Y. The frequently involved chromosomes in our group were 5 (in 6/7 patients), 8 and 17 (each in 4/7 patients) as previously reported [3]; anomalies of chromosomes 3, 11, 12, 16 and 21 were also frequent occurrences (each in 3/7 patients). Contrary to previous reports, chromosome 7 was seldom described in the abnormal genomic profiles of our group, most probably due to the low number of analyzed patients. Overall, from all of the chromosomes described by classical karyotyping as altered, 13 and 19 were not found to have aberrant genomic profiles, due to the fact that they were involved in structural rearrangements with no gains or losses. Array-CGH+SNP was extremely useful in CK, accurately describing the size, gene content and breakpoints for various aberrant regions. In addition, in four patients, microarrays detected the involvement of chromosomes not previously identified by cytogenetics. Moreover, in some patients, Array-CGH+SNP refined the size of the involved genomic region, for example, patient 39 (Supplementary Materials Table S1) with a large del(5)(q31q35) described by karyotyping that proved to be much smaller on the genomic profile (2 Mb, band 5q31.2), with the apparently deleted chromosome 5 being, in fact, structurally rearranged. The same patient had a CN-LOH of 9p24.3p13.1 that potentiated a *JAK2V617F* mutation to homozygous 19q12q13.12 and its deletion, leading to a homozygous *CEBPA* gene mutation. One patient (patient 19, Supplementary Materials Table S1) had a complex rearrangement of chromosome 8q with two deletions flanking an amplified region, 8q24.13q24.22 (with a copy number on array of 3–10) including *MYC. MYC* amplification is a common defect in AML [17,21]; the minimal commonly amplified region was defined by L’Abbate and spans 2.2 Mb, encompassing 15 RefSeq [41]. Our region significantly overlapped this critical region. The amplification of chromosome 21 was detected by microarray in three patients and by karyotyping in two patients, the latter showing complex rearrangements of this chromosomes (isochromosome 21, other structural rearrangements). One patient (patient 42, Supplementary Materials Table S1) had a high amplification level of chromosome 21 material with 7–8 copies described by microarray but with a normal copy number for the *RUNX1* gene. By contrast, the other two patients (patients 41 and 9, Supplementary Materials Table S1) had lower levels of amplification, and *RUNX1* gene deletions. This pattern of genetic defect was described previously [35].

### 3.3. Targeted Mutational Screening

Targeted NGS and *FLT3*-ITD screening revealed a total of 108 pathogenic or likely-pathogenic variants in 86.7% of patients (39/45), a mutational rate similar to that described in other reports [2,42,43]. The number of variants per patient varied between 0 and 5 with a median of two mutations per sample. Missense mutations accounted for the majority (70.9%) of the identified mutations, followed by nonsense and frameshift variants (10.9% each), splice-site mutations (5.5%) and in-frame insertions (1.8%). Eight mutations were bi-allelic, four homozygous and four double-heterozygous, with *TP53* being the most frequently targeted gene.

Ninety-four variants affected 45 recurrent codons/loci, while 14 newly described variants were non-recurrent in our group. The number of unreported mutations was high in *TET2* (5/9), *DNMT3A* (4/9), *CEBPA* (2/4) and *TP53* (2/5), and only one new variant was observed in *RUNX1*. The majority of the unreported mutations were missense (7/14) but truncating (three frameshift, two nonsense), splice-site (one) and indel (one) mutations were also detected.

We found five unreported mutations in *TET2* with damaging predictions. These variants were mainly missense (4/5) and occurred predominantly within an evolutionary conserved domain, CD1. Our findings are in line with those reported by Weissmann et al. [44]. 

The somatic unreported mutations in *DNMT3A* were located between exons 15 and 23 and were predicted to affect the methyltransferase domain hotspot for mutations in AML [45]; truncating variants were present in three out of four patients. 

Both patients with bi-allelic *CEBPA* had a combination of one frequently reported and one new variant; the newly described mutations followed the well-known pattern of pathogenic sequence variation (N-terminal frameshift and C-terminal in-frame deletion/duplication mutations) [46,47]. 

We identified two missense mutations located in the DNA-binding domain of *TP53*. These mutations were not reported in hematological malignancies but found in solid tumors (COSMIC). 

One unreported canonical splice-site mutation was found in the *RUNX1* gene. The variant was located at +1 between exons 6 and 7 corresponding to the C-terminal of the runt-homology domain (RHD) of the protein that is responsible for DNA-binding and interaction with CBFβ [48].

Both *FLT3-ITD* and *TK2* mutations were investigated in our group. *FLT3-ITD* mutations were detected in 23% of the tested patients (10/44). One patient was homozygous for the mutation due to a CN-LOH of 13q; all the other patients had heterozygous *FLT3-ITDs*. One patient had two mutant alleles with different ITD sizes. Two patients were positive for recurrent *FLT3-TK2* mutations; none of the patients were double mutant for ITD and TK2 point mutations in the *FLT3* gene. In the present study, *FLT3* mutations were identified in 26.66% (12/45) of the patients, a frequency comparable with the previously described rate (~30%) [49,50,51,52].

The variants with clinical significance identified by NGS were located in 15 out of 19 of the studied genes. Overall, the most prevalent genes targeted by mutations in our study (>20% of patients, each) were *NPM1*, *DNMT3A*, *FLT3* (ITD mutations included) and *NRAS* (Table 1), in agreement with other studies [4,43]. *NPM1*, *DNMT3A*, and *FLT3* mutations occurred with the highest frequency; these data are in line with previous reports [2,43,53,54]. No mutations were observed in *ASXL1,* although this gene is frequently targeted by sequence alterations [4,55].

#### 3.3.1. NGS in NK Group 

NGS mutational screening performed in the NK group (28 patients) revealed pathogenic variants in 85.7% (24/28), the frequently mutated genes being *NPM1* (44.44%), *DNMT3A* (42.9%), *NRAS* (35.7%), *FLT3-ITD/TK2* (32.1%), *TET2* and *IDH1* (21.4% each) [56]. In the *NPM1* mutation group, frequent associations were observed with the following genes: *DNMT3A* (55.5%), *FLT3* (44.4%), *NRAS* (38.8%), *TET2* (22.2%), *IDH1* (22%) and *PTPN11* (11.1%). Six out of 10 patients who shared *DNMT3A* and *NPM1* mutations also presented *FLT3* variants, a frequently reported association [4,13].

Bi-allelic *CEBPA* mutations co-occurred with *GATA2* variants (bi-allelic or single mutations) and *NRAS* in two NK patients. Another two patients presented C-terminal mutations in CEBPA: a heterozygous mutation in a patient with intermediate cytogenetics and a homozygous variant due to the CN-LOH on 19q13 in a patient with adverse cytogenetics. Bi-allelic *CEBPA* mutations were reported in 11–13% of NK patients [47,57]; in our cohort, bi-allelic *CEBPA* mutations were observed in 7% (2/30) of the patients with NK. 

#### 3.3.2. NGS in the Intermediate Cytogenetics Group 

In the intermediate cytogenetics group (six patients), variants in *NPM1* (two patients), *IDH2* (two patients), *RUNX1* (two patients), *PTPN11* (one patient) and mono-allelic *CEBPA* (one patient) were observed with no preferential mutation co-occurrence. One patient with *RUNX1* mutation had trisomy 13: two genetic lesions known to be strongly associated [58].

#### 3.3.3. NGS in the Adverse Cytogenetics Group 

*TP53* mutations were highly prevalent in the cytogenetic adverse group with a frequency of 44% (6/9 patients), in contrast with the NK and intermediate cytogenetics groups where no *TP53* mutations were detected [59]. Three patients had bi-allelic *TP53* alterations: homozygous mutations as the result of CN-LOH (one patient) and chromosome 17p13 deletion (one patient) and a double heterozygous mutation (one patient). Another recurrently mutated gene was *DNMT3A,* observed in 33% of patients (3/9 patients); mutations in *FLT3, NRAS, TET2, IDH2, PTPN11, BRAF* and *JAK2* were each observed in one patient only.

In our study, three patients had *KMT2A* gene alterations (two translocations and one PTD). The mutational burden of *KMT2A*-positive patients was low with no sequence variants (2/3) and two variants in preleukemic genes (1/3). 

The mutational spectrum in our study group was overall similar to that in previous reports regarding the mutation frequency, preferential associations and mutual exclusivity. One exception was the absence of variants in *ASXL1,* which could be explained by the small cohort size. Another exception, an unexpected co-occurrence of *KMT2A* translocation and *DNMT3A* mutation (R882), was identified; alterations of these two genes involved in epigenetic regulation are strongly considered to be mutually exclusive [4,56].

The mutational screening was highly informative for two patients with no available cytogenetics and normal genomic profiles by array-CGH+SNP. Three and four mutations, respectively, were detected, with the co-occurrence of *NPM1, DNMT3A*and *FLT3* variants in both patients.

### 3.4. Clinical Impact of Comprehensive Cytogenetic and Molecular Investigation 

In our patient group, the cytogenetic and genetic investigation allowed WHO diagnostic classification in 75% (36/48) of the patients. NGS proved to be the most informative test within our integrated strategy, 26 out of 36 patients with WHO diagnostic harboring mutations in entity-defining AML genes (20 patients with *NPM1* mutation, two patients with bi-allelic *CEBPA* variants, four patients with *RUNX1* mutations). 

The genetic risk evaluation according to ELN 2017 [11] allowed patient stratification in the following categories: favorable risk—19 patients, intermediate risk—15 patients and adverse risk—14 patients. Prognostic stratification of the patients with complex karyotype relied solely on cytogenetics; however, CMA and NGS allowed a better molecular characterization. The risk category was further refined, by our integrated approach, in two out of six patients with intermediate cytogenetics in whom NGS revealed mutations in the *RUNX1* gene. NGS had a major clinical impact in the normal karyotype AML group by allowing risk stratification of 82% (23/28) of the patients into one of the following categories: adverse risk, two patients with *RUNX1* mutations, and favorable risk, with 17 patients with mutated *NPM1* (without *FLT3*-ITD or with low *FLT3*-ITD allelic ratio) and two patients with bi-allelic *CEBPA* variants.

## 4. Conclusions

In summary, our results show that by the use of an integrated approach consisting of cytogenetics, array-CGH+SNP and targeted NGS, at least one genetic anomaly was detected in all the patients. In normal karyotype AML, 86% of the patients had gene mutations and 25%, genomic imbalances. In the groups with abnormal cytogenetics, the prevalence of gene mutations was 87.5%, while genomic profiling aided the accurate description of molecular lesions and revealed defects undetected by other methods. Thus, the integration of multiple investigation strategies increases the detection yield of genetic defects with potential clinical relevance. 

## Figures and Tables

**Table 1:** Frequency of mutated genes in our mutational screening group (45 patients).

Targeted Genes	Frequency % of Patients (Number)	Mutation Subtype	Frequency % of Patients (Number)
*NPM1*	44.44 (20)	Subtype A	33.33 (15)
		Subtype B	4.44 (2)
		Subtype D	6.67 (3)
*DNMT3A*	42.22 (19)	R882	24.44 (11)
		Non-R882	17.78 (8)
*FLT3*	26.66 (12)	ITD	22.22 (10)
		TK2 point mutations	4.44 (2)
*NRAS*	24.44 (11)		
*TET2*	15.56 (7)	BI	4.44 (2)
		MO	11.11 (5)
*IDH1*	13.33 (6)		
*IDH2*	11.11 (5)		
*TP53*	8.89 (4)	HO (deletion or CN-LOH)	4.44 (2)
		BI	2.22 (1)
		MO	2.22 (1)
*CEBPA*	8.89 (4)	BI	4.44 (2)
		MO	2.22 (1)
		HO (deletion)	2.22 (1)
*PTPN11*	8.89 (4)		
*RUNX1*	8.89 (4)		
*GATA2*	4.44 (2)	BI	2.22 (1)
		MO	2.22 (1)
*KRAS*	4.44 (2)		
*BRAF*	2.22 (1)		
*JAK2*	2.22 (1)		
*ASXL1*	−		
*CBL*	−		
*KIT*	−		
*WT1*	−		

BI—bi-allelic; MO—mono-allelic; HO—homozygous.

## Data Availability

The main data generated and analyzed in our study are included in this article and the Supplementary Files. The microarrays and sequencing data analyzed in our study are available from the corresponding author on reasonable request.

## Supplementary Materials

The following are available online at https://www.mdpi.com/article/10.3390/genes12060846/s1, Supplementary Table S1: Mutational screening, genomic profiling and cytogenetic testing detailed results.
